# *CDH1* methylation analysis in invasive lobular breast carcinomas with and without gene mutation

**DOI:** 10.1007/s00428-024-03814-8

**Published:** 2024-05-07

**Authors:** Silvia González-Martínez, Viera Horvathova Kajabova, Belén Pérez-Mies, Irene Carretero-Barrio, Tamara Caniego-Casas, David Sarrió, Gema Moreno-Bueno, María Gión, José Perez-García, Javier Cortés, Bozena Smolkova, José Palacios

**Affiliations:** 1 “Contigo Contra el Cáncer de la Mujer” Foundation, 28010 Madrid, Spain; 2 Molecular Pathology of Cancer Group, Ramón y Cajal Health Research Institute (IRYCIS), 28034 Madrid, Spain; 3 Centre for Biomedical Research in Cancer Networks (CIBERONC), Carlos III Health Institute, 28029 Madrid, Spain; 4 Department of Molecular Oncology, Cancer Research Institute, Biomedical Research Center of the Slovak Academy of Sciences, Dubravska Cesta 9, 84505 Bratislava, Slovakia; 5 Department of Pathology, Ramón y Cajal University Hospital, 28034 Madrid, Spain; 6 Faculty of Medicine, University of Alcalá, 28801 Madrid, Spain; 7 Department of Biochemistry, Universidad Autónoma de Madrid (UAM), Instituto de Investigaciones Biomédicas ‘Alberto Sols’, Conexión Cáncer (UAM-CSIC), 28029 Madrid, Spain; 8 MD Anderson Internacional Foundation, 28033 Madrid, Spain; 9 Department of Medical Oncology, Ramón y Cajal University Hospital, 28034 Madrid, Spain; 10 International Breast Cancer Center (IBCC), Pangaea Oncology, Quiron-salud Group, 08017 Barcelona, Spain; 11 Medica Scientia Innovation Research, 08007 Barcelona, Spain; 12 Medica Scientia Innovation Research, Ridgewood, NJ 07450 USA; 13 Department of Medicine, Faculty of Biomedical and Health Sciences, European University of Madrid, 28670 Madrid, Spain; 14 IOB Institute of Oncology Madrid, Hospital Beata María Ana, Madrid, Spain

**Keywords:** Breast cancer, Lobular carcinomas, *CDH1*, DNA methylation

## Abstract

The proposed role of *CDH1* (E-cadherin gene) methylation as a mechanism of gene inactivation in invasive lobular carcinoma (ILC) remains inconclusive. For many years, *CDH1* promoter hypermethylation has been regarded as a mechanism for gene inactivation in ILC. However, this assumption has primarily relied on non-quantitative assays, which have reported *CDH1* methylation frequencies ranging from 26 to 93% at CpG sites within the island region. Few studies employing quantitative methods and covering CpG island shores, regions of relatively low CpG density situated proximal to conventional promoter CpGs, have been conducted, revealing lower percentages of methylation ranging from 0 to 51%. Therefore, using the quantitative pyrosequencing method, we examined *CDH1* methylation in the island region and shores in E-cadherin deficient ILC cases (15 with *CDH1* mutation and 22 non-mutated), 19 cases of invasive breast carcinomas non-special type (IBC-NSTs), and five cases of usual ductal hyperplasia (UDH). Our analysis revealed *CDH1* methylation frequencies ranging from 3 to 64%, with no significant increase in methylation levels in any group of ILCs (median = 12%) compared to IBC-NST (median = 15%). In addition, considering the poorly studied association between the number of tumor-infiltrating lymphocytes (TILs) and *CDH1* methylation in breast cancer, we undertook a thorough analysis within our dataset. Our findings revealed a positive correlation between *CDH1* methylation and the presence of TILs (*r* = 0.5; *p*-value < 0.05), shedding light on an aspect of breast cancer biology warranting further investigation. These findings challenge *CDH1* methylation as a *CDH1* inactivation mechanism in ILC and highlight TILs as a potential confounding factor in gene methylation.

**Supplementary Information:**

The online version contains supplementary material available at 10.1007/s00428-024-03814-8.

## Introduction

Invasive lobular carcinoma (ILC) is the second most common type of invasive breast cancer, accounting for around 10–15% of all cases. ILC is characterized by its unique growth pattern. The key molecular hallmark is the loss of the epithelial cell-cell adhesion molecule E-cadherin, encoded by *CDH1*, which occurs in 85% of ILC [1]. The molecular mechanisms involved in the decrease or even loss of this protein vary. The *CDH1* gene can be inactivated by mutations (50–60% cases) and loss of heterozygosity [1].

For many years, *CDH1* promoter hypermethylation has been accepted as a mechanism for gene inactivation in ILC. This assumption largely stems from non-quantitative assays, predominantly methylation-specific PCR (MSP), which reported *CDH1* methylation frequencies ranging from 26 to 93% [2–7]. However, it has been demonstrated that MSP can yield a significant number of false-positive results [8].

The comprehensive TCGA study by Ciriello et al. [9] challenged the hypothesis of frequent *CDH1* methylation in ILC. Analyzing 111 ILCs via Illumina Infinium DNA methylation HumanMethylation 27 (HM27) and HumanMethylation 450 (HM450) platforms, the authors unveiled similar *CDH1* methylation patterns in ILCs and invasive breast carcinomas non-special type (IBC-NSTs), the latter characterized by preserved E-cadherin expression. While the study encompassed both *CDH1* wild-type and mutated ILCs, it lacked a dedicated analysis of methylation data stratified by mutation status or *CDH1* mRNA expression, positive in 13% of samples. More recently, Alexander et al. [10] also failed to identify significant *CDH1* promoter methylation in nine ILC cases exhibiting varying levels of E-cadherin expression through methylation EPIC BeadChip 850K array analysis.

It is noteworthy that a variable that has been underexplored in *CDH1* methylation studies in breast cancer is the role of tumor-infiltrating lymphocytes (TILs), despite Lombaerts et al. [5] first describing lymphocyte infiltration as a factor to consider in 2004 due to its potential influence on the detection of *CDH1* promoter methylation in breast tumors.

In light of these discoveries, we hypothesized that if *CDH1* methylation contributes to *CDH1* gene inactivation in ILCs, it would be more prevalent in tumors lacking E-cadherin expression and devoid of *CDH1* mutation. In this selected group of cases, *CDH1* methylation could present itself as a viable alternative mechanism for inducing inactivation, complementing the role typically fulfilled by gene mutations. To test this hypothesis and to evaluate the possible impact of the abundance of TILs on methylation results, we analyze a group of ductal and lobular carcinomas with different *CDH1* mutational status and TIL abundance by pyrosequencing.

## Methods

The study received approval from the Local Ethics Committee (Ramón y Cajal Research Ethics Committee reference 223/18). A total of 61 cases were selected from the Pathology Department of Ramón y Cajal University Hospital (Madrid, Spain). The selection process for the studied cases was based on data availability from prior sequencing studies conducted in our laboratory, along with more recent cases diagnosed within the past year through the Pathology Department. These latter cases underwent thorough DNA sequencing as part of their evaluation process. Clinical data were obtained from clinical databases. Histological evaluation, immunohistochemistry, and sequencing were carried out as previously reported [11].

TIL evaluation was conducted in regions where DNA was extracted for methylation analysis, following the recommendations of the TILs Working Group [12].

Genomic DNA (2 μg) from all tumors was used for sodium bisulfite treatment using the EpiTect Bisulfite kit (Qiagen). This approach ensures the complete conversion of unmethylated cytosine to uracil, enabling the detection of methylated CpGs. Four sets of primers were designed, covering 18 CpG dinucleotides in the regulatory regions of the gene *CDH1*–N-shore, CpG Island, and S-shore, using the PyroMark Assay Design 2.0 software (Qiagen) (Supplementary Table 1). Quantitative pyrosequencing was employed to assess the DNA methylation of these regulatory regions. PCR amplification was conducted with PyroMark PCR Kit (Qiagen) as per the manufacturer’s instructions. Pyrosequencing was performed using the PyroMark Gold Q24 Reagents (Qiagen) on a PyroMark Q24 platform. Data analysis utilized the PyroMark Q24 2.0.6. software (Qiagen). Median methylation values for each CpG were compared among the three groups of tumors (Kruskal-Wallis or ANOVA test). To examine differences in methylation levels across studied regions, the median of the mean methylation values of the CpG sites per region were compared among groups (Kruskal-Wallis or ANOVA test). All statistical tests and plots were conducted using the R software.

## Results and discussion

To ascertain the prevalence of *CDH1* methylation in ILCs characterized by both the absence of *CDH1* mutation in the exonic region and E-cadherin expression, we conducted quantitative pyrosequencing on a cohort of 22 ILC cases that had undergone comprehensive massive parallel sequencing, revealing a lack of *CDH1* mutations and complete E-cadherin expression absence [11, 13, 14]. For comparative purposes, we analyzed 15 ILC cases with *CDH1* mutations and full E-cadherin expression loss, along with 19 IBC-NSTs marked by preserved E-cadherin expression and no *CDH1* mutation and five cases of usual ductal hyperplasia (UDH). The main clinicopathological and molecular data of the patients are presented in Supplementary Table 2. In summary, tumors were diagnosed in patients aged between 36 and 92 years old (median = 63 years old). Concerning the histological type, 30% of the ILC tumors exhibited pleomorphic lobular characteristics. In terms of molecular subtype, 86% of the tumors were luminal (32 ILC and 16 IBC-NST), 12% were triple-negative (4 ILC and 3 IBC-NST), and 2% were luminal HER2+ (1 ILC).

Pyrosequencing is a high-resolution method for the detection of DNA methylation and provides quantitative information for each CpG site under study, allowing for the control of bisulfite conversion efficiency. Pyrosequencing is the technique with the best reproducibility (even higher than methylation array) and can work well even on minute amounts of highly fragmented DNA [15].

**Fig. 1:**
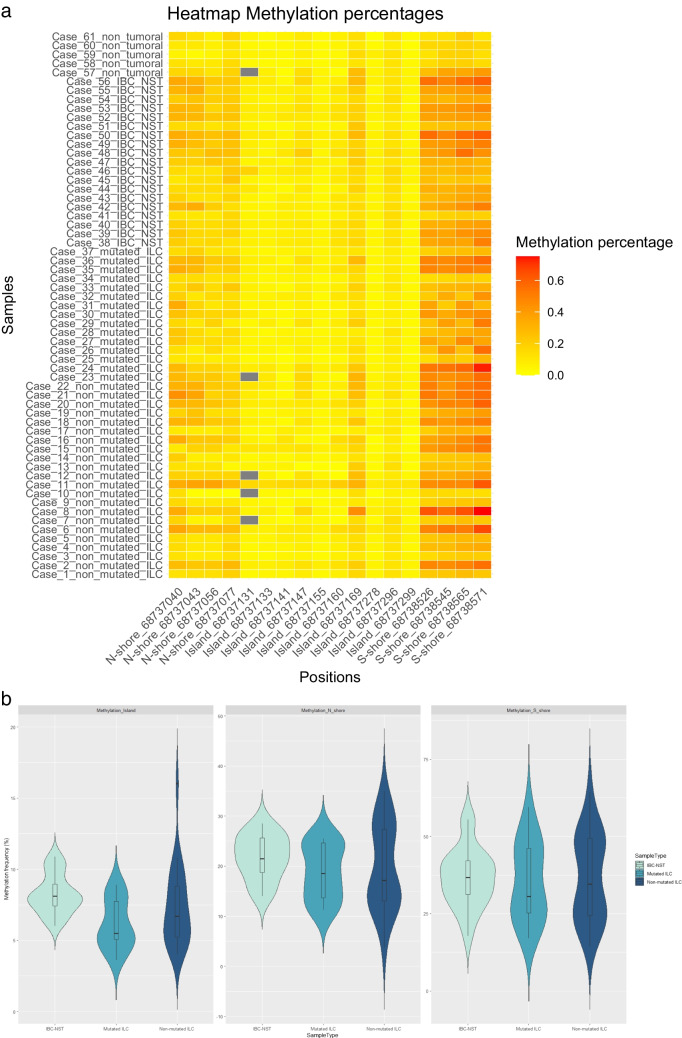
**a** DNA methylation status of sequenced CpGs sites aligning with the *CDH1* gene. Grey boxes = data not available. **b** Violin plots depicting methylation data across each tumor subgroup within the respective region

Within this cohort of 56 primary tumors, we comprehensively examined the methylation status of 18 CpG dinucleotides situated in the CpG island (103 bp) of the *CDH1* gene and in the Northern and Southern shore (N-shore, S-shore) regions. Analyzing the differences in methylation frequency according to molecular phenotype (triple-negative vs. luminal), we did not observe statistically significant differences, neither in general nor in any of the methylation zones (N-shore, island, and S-shore) (*p*-value > 0.05). The median methylation frequency in ILCs was 12%, while in IBC-NSTs, it was 15% (*p*-value > 0.05). The CpG island region encompassed the majority of sites explored in prior MSP studies as well as four CpGs as scrutinized by Ciriello et al. [9] and five CpGs by Alexander et al. [10] through methylation arrays (Supplementary Tables 3, 4, 5). Notably, the observed methylation values in the CpG island were generally modest (ranging between 3 and 18%) (Fig. 1a). CpG methylation values statistically differ among the studied groups for CpG sites at positions 68737141, 68737278, 68737296, and 68737299 located in the CpG island region. Interestingly, these CpGs exhibited slightly heightened methylation levels in IBC-NSTs (*p*-value < 0.05) (Supplementary Fig. 1 B-E). Furthermore, there were significant differences in methylation levels in the whole island region between the group of mutated ILCs and IBC-NSTs, the latter being higher (Fig. 1b).

Tissue- and cancer-specific differentially methylated regions can occur not only within CpG islands themselves but also within CpG island shores, regions of relatively low CpG density, situated proximal to conventional promoter CpGs (up to 2 kb distant). This suggests the potential involvement of shore methylation in tissue differentiation, epigenetic reprogramming, and cancer [16]. Intriguingly, the analysis of *CDH1* shore methylation has not been analyzed in MSP studies (Supplementary Table 3). Therefore, we extended our primer design to CpGs located in both N-shore and S-shore (Supplementary Table 3). Methylation levels in these regions were, in general, higher than in the CpG island (ranging from 4 to 35% and 14 to 64%, respectively) (Fig. 1a). There were significant differences between the studied groups for CpG site at position 68737077 in terms of CpG site-specific comparisons (Supplementary Fig. 1A), but there were no significant differences for whole region assessments (Fig. 1b).

We further examined these CpG sites in the five non-tumoral tissue samples, revealing lower methylation percentages compared to tumor samples across all regions (Fig. 1a).

In an effort to corroborate our findings, we compare our results with those reported by Ciriello et al. [9] and Alexander et al. [10]. Unfortunately, the available datasets from Ciriello et al. lack explicit specification of methylation beta values corresponding to the individual probes, offering a graphical overview instead. Since they did not make a differential analysis between the methylation status of *CDH1*-mutated and non-mutated cases, we compared the methylation frequencies at each CpG site for both ILC groups combined (with and without *CDH1* mutation). Conversely, the dataset provided by Alexander et al. [10] allowed us to compare methylation levels in ILCs according to *CDH1* mutation status, although the small number of cases lacking *CDH1* mutation (*n* = 4) was a significant limitation of data reproducibility. In general, we observed similarity in methylation levels when compared to those outlined by Ciriello et al. [9], while we demonstrated lower methylation levels in contrast to those observed in the study by Alexander et al. [10] (Supplementary Tables 4 and 5). Furthermore, Fridrichova et al. [17] reported *CDH1* methylation levels assessed by pyrosequencing across seven identical CpGs situated within the CpG island among 24 ILC cases, 178 invasive ductal carcinoma, and four other breast cancer patients. Although the mutational status of ILC cases was not assessed in this study, consistent with our current results, there were no disparities in DNA methylation across these groups, and the average value in tumors and paired lymph node metastasis remained below 10.5% [17].

**Fig. 2:**
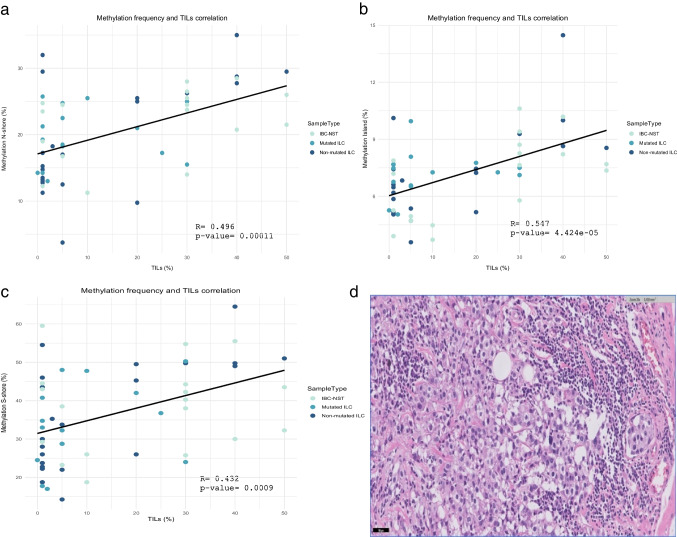
**a**, **b**, **c** Correlation between *CDH1* methylation frequency and percentage of TILs across N-shore, island, and S-shore regions. **d** Hematoxylin-eosin staining displaying the percentage of TILs (40%) in non-mutated ILC case number 11

While we did not observe substantial differences in *CDH1* methylation across diverse tumor subtypes, noteworthy instances of elevated methylation were noted in selected tumors, such as cases 2, 8, 11, 50, or 56, among others. The relevant aspect to be considered is that *CDH1* methylation can occur in TILs, thereby introducing a confounding element that can lead to false positive outcomes, particularly when using MSP [5]. To confirm this hypothesis, we conducted a correlation analysis between TILs and methylation levels across different CpGs, unveiling a modest yet statistically significant correlation between TILs and methylation levels across all examined regions (*p*-value < 0.05) (Fig. 2). In our observations, the percentage of TILs was found to be slightly higher in the IBC-NST group (median = 30%), exhibiting a statistically significant difference compared to the mutated-ILC group (median = 5%) (*p*-value < 0.05). This disparity could influence the higher methylation percentage observed in the IBC-NST group across all studied regions (Fig. 1; Supplementary Fig. 1). Additionally, UDH displayed low methylation percentages (median 11%, 6%, and 16% methylation in N-shore, Island, and S-shore regions, respectively) (Fig. 1a), alongside a considerably low TILs percentage ranging from 0 to 10% (median = 4%), which could influence the low methylation levels.

In conclusion, our findings, facilitated by high-resolution quantitative detection methodology, indicated that the frequency and extent of *CDH1* gene methylation in ILCs are not higher than those observed in IBC-NSTs. This result held true irrespective of the presence or absence of *CDH1* mutations, thereby challenging the notion of *CDH1* methylation as a pervasive mechanism for *CDH1* gene inactivation. Moreover, our analysis suggested the potential impact of TIL abundance on *CDH1* methylation analysis. Importantly, the conspicuous loss of E-cadherin in the non-mutated ILC subgroup might be driven by mechanisms beyond DNA methylation. The intricate interplay of additional genetic and epigenetic mechanisms, along with non-genetic determinants such as cellular signaling pathways, environmental factors, and cellular context, holds promise in shedding light on alternative mechanisms to the loss of *CDH1* for the lobular phenotype [18].

### Supplementary information

ESM 1 (XLSX 917 kb)

## Data Availability

Additional information on this article can be found in supplementary material.

## Abbreviations

ILC: Invasive lobular carcinoma
IBC-NSTs: Invasive breast carcinoma non-special type
TILs: Tumor-infiltrating lymphocytes
MSP: Methylation-specific PCR
HM27: HumanMethylation 27
HM450: HumanMethylation 450
N-shore: Northern shore
S-shore: Southern shore

## Declarations

### Disclaimer

The funders played no role in study design, data collection, analysis and interpretation of data, or the writing of this manuscript.
